# Learners’ Perspectives of Professionalism: Protocol for a Mixed Methods Systematic Review

**DOI:** 10.2196/37473

**Published:** 2022-08-25

**Authors:** Nagina Khan, Walther van Mook, Subodh Dave, Sohyun Ha, Joshua Sagisi, Nicole Davi, Chantel Aftab, Sucheta Tiwari, Marie Hickman, Wolfgang Gilliar

**Affiliations:** 1 Department of Osteopathic Medicine Touro University Nevada Henderson, NV United States; 2 Association of University Teachers of Psychiatry London United Kingdom; 3 Department of Intensive Care Medicine Maastricht University Medical Centre Maastricht Netherlands; 4 School of Health Professions Education Faculty of Health, Medicine, and Life Sciences Maastricht University Maastricht Netherlands; 5 Maastricht UMC+ Academy Maastricht Netherlands; 6 Department of Osteopathic Medicine Derbyshire Healthcare NHS Foundation Trust Derbyshire United Kingdom; 7 Department of Osteopathic Medicine University of Bolton Bolton United Kingdom; 8 Royal College of Psychiatrists London United Kingdom; 9 East London NHS Foundation Trust London United Kingdom

**Keywords:** professionalism, undergraduate medical education, medical school, medical education, medical curriculum, teaching methods, teaching, medical students, student, undergraduate, convergent integrated synthesis, integrated synthesis, curriculum, recommendation, learner, perspective, review

## Abstract

**Background:**

Professionalism has come to be associated with competence in medical education, with the habitual and judicious use of communication, knowledge, technical skills, clinical reasoning, emotions, values, and reflection in daily practice for the benefit of the individual and community being served. Recent studies indicate students should have the opportunity to observe the application of knowledge and skills by their mentors to improve patient health and safety. A noticeable detail that needs implementation into the curriculum is the inclusion of student perspectives. This review will explore students’ understanding and experience of professionalism in undergraduate medical education (UME).

**Objective:**

This paper presents the protocol for a review that aims to develop an integrated synthesis of qualitative and quantitative studies resulting in recommendations for medical school curricula to incorporate the learners’ perspectives in teaching professionalism in UME.

**Methods:**

We will take an integrated approach to synthesis. Data will be extracted from the included studies, and quantitative data will be “qualitized.” PubMed (Medline), Embase, PsycInfo, and ERIC (Education Resources Information Center) will be searched for studies published in English from 2010 to 2021. Studies will be screened and critically appraised for methodological quality using the Mixed Methods Appraisal Tool by 2 researchers, with disagreements resolved by a third researcher. Qualitative, quantitative, and mixed methods studies will be considered. Our population of interest is undergraduate medical students; hence, studies on medical residents and graduate medical students will be excluded. We will consider studies that explore how concepts of professionalism are understood, experienced, and taught in undergraduate medicine and on how medical students understand and develop the identified constructs of professionalism.

**Results:**

This study is in the screening phase; therefore, no results are available at this time. However, we had initiated the searches, screening, and are currently in the critical appraisal stage. We will commence preparation to clean and convert the data for coding in July 2022, and analysis will be ongoing from the end of July 2022 until submission for publication in November 2022.

**Conclusions:**

This research will contribute to the student perspectives on professionalism in medical education literature. The findings will aid in the creation of a checklist to guide the development of a curriculum on professionalism in UME.

**International Registered Report Identifier (IRRID):**

PRR1-10.2196/37473

## Introduction

### Background

Professionalism has become an important topic in medical education with growing recognition of the importance of medical students and doctors in developing excellence in professionalism [1]. The Association of American Medical Colleges states that physicians must be altruistic, knowledgeable, skillful, and dutiful [2]. Among the 6 general competencies listed by the Accreditation Council for Graduate Medical Education are “interpersonal and communication skills that result in effective information exchange and teaming with patients, their families, other health professionals” and “professionalism, as manifested through a commitment to carrying out professional responsibilities, adherence to ethical principles, and sensitivity to a diverse patient population” [3,4]. Competence in these areas not only necessitates that training programs support the development of competence but also that they evaluate students’ professionalism. The American Board of Internal Medicine certification program contains issues associated with ethics and professionalism [5]. These efforts accentuate the importance of training and assessing professionalism [6]. Professionalism remains a recognized core competency of doctors [7], implying that the classification and definition of professionalism are also subjected to contextual and temporal changes [8]. Professionalism is an important aspect of training and health systems; however, integration into teaching varies, from formal teaching to learning through nonintegrated methods such as the hidden curriculum. Two elements, namely context (environment) and learners’ perspectives, have shown to be important in undergraduate medical education (UME) because students training to be physicians need to develop a sense of consistency that contributes context specificity as well as resilience to working in complex, rapidly changing environments. The shifting debate in medical education emphasizes that education remains learner centered and should be guided by learner needs [9].

The perspective of learners is underrepresented in the literature, with few studies focusing on learners. In recent years, a number of investigations have presented evidence on how certain types of medical education, such as longitudinal integrated clerkships [1-3], can support professionalism and patient-centered approaches. However, there is still insufficient evidence as to how, why, and in what circumstances learners’ perspectives and engagement with professionalism are valuable. These data are central if we are to improve medical education for the development of professional physicians [4]. Learners, as active participants in their education, may provide unique insights into not only the intervention but also the context in which the learning occurs (context-environment) [10]. Therefore, we contend that identifying and integrating approaches from learners’ perspectives is important for supporting their understanding of working in complex environments. This gap could prove fundamental to the engagement of students with professionalism and for them to shift from simply enacting key competencies to embedding them in real environments through their own thought processes as learners to increase satisfaction in their work, career, and professional competence in ways they find acceptable and easy to understand.

Consequently, the aim of our work is to explore undergraduate medical students’ views toward professionalism education and to identify the barriers to and facilitators of integration in the undergraduate medical curriculum.

### Objective

The objective of this review is to develop an integrated synthesis of qualitative and quantitative research to derive recommendations for UME relevant to incorporating learners’ perspectives in the current teaching of professionalism in the curriculum. Specifically, we aim to:

- Identify learners’ perceptions of the potential barriers to or facilitators of professionalism;
- Identify learners’ experience with the most useful methods that are used to teach professionalism;
- Determine future priorities for the curriculum, considering the strengths and limitations of the complexity of individual cases and the changing health care environment.

## Methods

### Inclusion Criteria

#### Population

We will include studies on undergraduate medical students, and we will exclude studies on medical residents and graduate medical students.

#### Phenomena of Interest

We will explore studies on learners’ perspectives, attitudes, understanding, and experiences of professionalism in UME.

#### Context

We will consider studies that explore the concepts of professionalism and medical students’ understanding of and experiences with it, including a focus on the teaching methods that allow experiential learning and context. The settings will include community practices, hospitals, and academic settings.

#### Types of Studies

This review will consider qualitative, quantitative, and mixed methods studies.

Qualitative studies will include designs such as primary qualitative studies, underpinned with ethnography, phenomenology, and grounded theory; ethnographic interviews; narrative studies; and program evaluations. Unpublished studies will not be included.

Quantitative studies will be included if they are program evaluations.

Studies published in English (for easy access and ease of interpretation) and published from 2010 to 2021 will be included. Since contemporary health systems are changing, we will focus on current perceptions as related to student understandings and not the historical beginnings of professionalism.

### Study Design

The integrative review method (Textbox 1) was selected for this study to include a broad range of empirical studies (both qualitative and quantitative) [11-13]. This research design is appropriate as the research question is focused on the why and the how, as well as examining a contemporary phenomenon. This method is appropriate for our study because the integrative review reexamines, critiques, and synthesizes findings from separate but related research to develop new frameworks or perspectives about a specific phenomenon or topic [14]. An integrative review addresses a new or emerging topic as opposed to mature topics and provides an initial model rather than recreating previous models [12,14,15]. This method will be carried out via the following steps (also see Table 1):

- Create a search strategy with the specialist medical librarian;
- Search databases and remove duplicates from the retrieved articles using Mendeley Reference Manager (Mendeley Ltd);
- Screen the articles according to the inclusion and exclusion criteria using Rayyan software (Rayyan) [16];
- Conduct a critical appraisal using the Mixed Methods Appraisal Tool (MMAT) [17];
- Integrate both quantitative and qualitative data to focus on the same research question;
- Transform the data into a similar format (eg, “quantitized” or “qualitized” [18]);
- Comb narratively and present a narrative analysis of the findings.

Overall, the findings will elicit learners’ perspectives of professionalism and their experience of the most useful methods used during teaching.

**A summary of methodological approaches for convergent integrated mixed methods systematic reviews [13].**

Review design: Convergent integrated

Description: Involves data transformation that allows reviewers to combine quantitative and qualitative data

What is involved in the integration? Direct assimilation

Methods for integration: Content analysis, vote counting, thematic synthesis

**Table 1:** Outline of a mixed methods study.

Study component	Description
Qualitative	Construct a search strategy, retrieve articles, remove duplicates, and screen according to the inclusion criteria Critically appraise the articles included, code the data, and convert the data for analysis Analysis of data
Quantitative	Construct a search strategy, retrieve articles, remove duplicates, and screen according to the inclusion criteria Critically appraise the articles included, code the data, and convert the data for analysis Analysis of data
Mixed analysis	Qualitative data: identify themes Quantitative data: qualitize and identify themes Combine identified themes, draft a list of guidelines from the findings, and finalize the document and disseminate

### Information Sources

The databases searched include PubMed, Embase, PsycInfo, and ERIC (Education Resources Information Center).

### Search Strategy

The search strategy was created to retrieve both published and unpublished studies using the Peer Review of Electronic Search Strategies 2015 guideline [19]. An initial limited search of Embase, PubMed, PsycInfo, and ERIC was undertaken to identify articles on the topic. The keywords contained in the titles and abstracts of relevant articles, as well as the index terms used to describe the articles, were applied to develop a full search strategy for the 4 databases mentioned above (Multimedia Appendix 1) [20]. The search strategy, including all identified keywords and index terms, was adapted for each included information source.

### Study Selection

Following the search, all identified citations were collated and uploaded into Mendeley and duplicates were removed. The citations were exported to a systematic review software (Rayyan QCRI [16]). Titles and abstracts were then screened by 2 independent reviewers for assessment against the inclusion criteria for the review. In instances where reviewers did not agree, a third reviewer adjudicated on whether the article should be retrieved. At present, the full text of the selected citations has been assessed for inclusion by the reviewers. A third reviewer was asked to adjudicate if there is disagreement between the reviewers. Reasons for exclusion of full-text studies that do not meet the inclusion criteria were recorded and reported in the systematic review. The included articles will then be critically appraised using the MMAT [17]. The results of the search will be reported in full in the final systematic review and presented in a PRISMA (Preferred Reporting Items for Systematic Reviews and Meta-Analyses) flow diagram [21,22].

### Data Extraction

Data will be extracted from qualitative studies included in the review by 2 independent reviewers using customized Microsoft Word tables (Microsoft Corp) for recording and extracting data (see Tables S1 and S2 in Multimedia Appendix 2). The data extracted will include specific details about the population, context, culture, geographical location, study methods and findings, themes, and the phenomena of interest relevant to the review objective. Findings will be extracted and assigned a level of credibility. Any disagreements that arise between the reviewers will be resolved through discussion or with input from a third reviewer.

### Data Analysis

For qualitative and quantitative data to be integrated, data needs to be transformed either by converting qualitative data into quantitative data (ie, quantitizing) or by converting quantitative data into qualitative data (ie, qualitizing). This stage will be carried out by the 2 researchers qualified in qualitative research methods and verified by a third researcher. We will:

- Qualitize quantitative data;
- Extract the data and convert them into themes, categories, typologies, or narratives [18];
- Use thematic analysis [23,24] since it can be widely used across a range of epistemologies and research questions [25] (this technique can be used to distinguish, analyze, structure, explain, and describe themes found in a data set [26]).

### Ethical Considerations

This study does not involve the use of animal or patient data, or recruitment of human subjects. Consequently, the research conducted as part of this study presents minimal risk and fits one of the exempt review categories as defined by institutional review board regulations at Touro University Nevada.

## Results

This study is in the critical appraisal stage; therefore, no results are obtainable. At the writing stage of this protocol, we had initiated the searches for the review, which included search strategy development, removal of duplicate articles, and screening.

We received no external funding for this study.

## Discussion

### Complex Concepts

In this study, we will use mixed methods to generate a checklist of items for consideration in the process of developing a curriculum on professionalism for medical students. An integrative review design was selected for this study to allow for the use of both qualitative data and quantitative findings [27].

We anticipate most of the concepts of professionalism to be perceived as complex by learners. As the purpose of this study is to develop a checklist for guidance in generating a curriculum on professionalism, our key insights will be used to bring student understandings of professionalism into the formal teaching of this subject to improve the learning and training of physicians, to positively impact patient outcomes, and to reduce errors and risk.

We will investigate and focus on the understanding gained from using mixed methods to explore our study objectives. We contend that this combined focus on quantitative and qualitative data used collectively provides a rich understanding of professionalism teaching and would help create innovative academic curricula that contribute to teaching complex elements of professionalism.

We believe this will support our students in identifying early on not only with the profession but taking it one step further to also identify with the environments that they will likely be involved in when assessing, meeting, and treating patients. Additionally, our research aims to identify learners’ perceptions of actual or potential barriers to or facilitators of professionalism in those environments. Further research exploring such concepts and processes could be developed. Our international research team is committed to structuring contextual knowledge about professionalism, developing links that will exist longitudinally, and attempting to continually teach current findings on the topic of professionalism.

### Strengths and Limitations

The strength of this study includes the use of both quantitative and qualitative methods. Furthermore, the research team is made of medical students and medical educators who have been involved in this project from the beginning. The guidance checklist will be coproduced with input from medical students. The study is limited in that we will not have the added benefit of accessing any raw data (including transcriptions, reflective notes, and author insights about the context of the studies included) [28].

### Conclusion

We aim to create a checklist to guide the development of a curriculum on professionalism. This checklist will directly incorporate insights from student learners and will have detailed justifications and rationale for a curriculum on professionalism.

Our study will potentially have implications for learning, teaching, and future assessment of professionalism in medical education, health systems, and educational policies.

## Appendix

Multimedia Appendix 1 Search strategy.

Multimedia Appendix 2 Data extraction tables.

## Abbreviations

ERIC: Education Resources Information Center
MMAT: Mixed Methods Appraisal Tool
PRISMA: Preferred Reporting Items for Systematic Reviews and Meta-Analyses
UME: undergraduate medical education
