# Supplementary material for: Learners’ Perspectives of Professionalism: Protocol for a Mixed Methods Systematic Review
Source: JMIR Res Protoc. 2022 Aug 25;11(8):e37473. doi: 10.2196/37473 (PMC9459844; doi:10.2196/37473)
Supplement: Multimedia Appendix 1 [file resprot_v11i8e37473_app1.docx]

# Appendix 1: Search strategy

## Medline Search Strategy

|  | **Search terms** | **Number of references** |
| --- | --- | --- |
| 1 | exp PROFESSIONALISM/ | 1419 |
| 2 | "ETHICS, PROFESSIONAL"/ | 6959 |
| 3 | "EDUCATION, MEDICAL, UNDERGRADUATE"/ | 25253 |
| 4 | "EDUCATION, MEDICAL"/ | 58435 |
| 5 | exp "STUDENTS, MEDICAL"/ | 37040 |
| 6 | ("medical education" OR "medical teach*").ti,ab | 44109 |
| 7 | (student* OR undergrad*).ti,ab | 316378 |
| 8 | ("medical student*").ti,ab | 43963 |
| 18 | (professionalism).ti,ab | 7639 |
| 19 | (1 OR 2 OR 18) | 14992 |
| 20 | (4 OR 6) | 89186 |
| 21 | (5 OR 7 OR 8) | 324273 |
| 22 | (20 AND 21) | 23575 |
| 23 | (3 OR 22) | 44311 |
| 24 | (19 AND 23) | 1032 |
| 25 | 24 [DT 2010-2021] [Languages English] | 668 |

## Embase Search Strategy

|  | **Search terms** | **Number of references** |
| --- | --- | --- |
| 26 | PROFESSIONALISM/ | 9752 |
| 27 | (professionalism).ti,ab | 9314 |
| 28 | (26 OR 27) | 14676 |
| 29 | exp "MEDICAL EDUCATION"/ | 330962 |
| 30 | exp "MEDICAL STUDENT"/ | 74913 |
| 31 | ("medical education" OR "medical teach*").ti,ab | 50423 |
| 32 | (student* OR undergrad*).ti,ab | 413063 |
| 33 | ("medical student*").ti,ab | 57918 |
| 34 | (29 OR 31) | 340742 |
| 35 | (30 OR 32 OR 33) | 433111 |
| 36 | (34 AND 35) | 80285 |
| 37 | (28 AND 36) | 2161 |
| 38 | 37 [DT 2010-2021] [English language] | 1516 |

## PsycInfo Search Strategy

|  | **Search terms** | **Number of references** |
| --- | --- | --- |
| 39 | PROFESSIONALISM/ | 3958 |
| 40 | (professionalism).ti,ab | 4473 |
| 41 | (39 OR 40) | 6620 |
| 42 | exp "MEDICAL EDUCATION"/ | 24925 |
| 43 | "MEDICAL STUDENTS"/ | 13795 |
| 44 | ("medical education" OR "medical teach*").ti,ab | 7635 |
| 45 | (student* OR undergrad*).ti,ab | 591980 |
| 46 | ("medical student*").ti,ab | 13649 |
| 47 | (42 OR 44) | 26934 |
| 48 | (43 OR 45 OR 46) | 593557 |
| 49 | (47 AND 48) | 13109 |
| 50 | (41 AND 49) | 524 |
| 51 | 50 [DT 2010-2021] [Languages English] | 353 |

## ERIC Search strategy

professionalism and medical and (student or undergraduate), limit to last 10 years

76 references retrieved

**Total references retrieved = 2,613**

**Removed duplicates = 1,858 references**
