# Supplementary material for: Learners’ Perspectives of Professionalism: Protocol for a Mixed Methods Systematic Review
Source: JMIR Res Protoc. 2022 Aug 25;11(8):e37473. doi: 10.2196/37473 (PMC9459844; doi:10.2196/37473)
Supplement: Multimedia Appendix 2 [file resprot_v11i8e37473_app2.docx]

# Appendix 2: Data Extraction Tables

## Table S1. Contextual information for the selected studies

|  | **Study ID** | **Source**  Title Author(s) Year of publication | **Aims**  Aims/purpose | **Context**  Type of study  Country of origin  Setting | **Data collection**  Methods | **Sampling and participants**  population  Sample size | **Findings from the studies** |
| --- | --- | --- | --- | --- | --- | --- | --- |
| 1. |  |  |  |  |  |  |  |
| 2. |  |  |  |  |  |  |  |
| 3. |  |  |  |  |  |  |  |
| 4. |  |  |  |  |  |  |  |
| 5. |  |  |  |  |  |  |  |

## Table S2. Data extraction: Identifying themes and constructs

|  | **Themes** | **Quotes from the studies in the review** | **Themes from the studies in the review** | **The synthesis:**  **translating the findings** |
| --- | --- | --- | --- | --- |
|  |  |  |  |  |
| **Subthemes** |  |  |  |  |
|  |  |  |  |  |
|  |  |  |  |  |
|  |  |  |  |  |
| **Subthemes** |  |  |  |  |
|  |  |  |  |  |
|  |  |  |  |  |
|  |  |  |  |  |
|  |  |  |  |  |
| **Subthemes** |  |  |  |  |
|  |  |  |  |  |
|  |  |  |  |  |
|  |  |  |  |  |
